# Exploring the co-occurrence of depression, anxiety and insomnia symptoms, diagnoses and treatments in primary care: observational study using UK primary care data

**DOI:** 10.1192/bjo.2024.20

**Published:** 2024-04-18

**Authors:** Danielle Nimmons, Juan Carlos Bazo-Alvarez, Christina Avgerinou, Joseph Hayes, David Osborn, Claudia Cooper, Irene Petersen, Kate Walters

**Affiliations:** Research Department of Primary Care and Population Health, University College London, UK; Division of Psychiatry, University College London, UK; Centre of Psychiatry and Mental Health, Wolfson Institute of Population Health, Queen Mary University London, UK

**Keywords:** Anxiety or fear-related disorders, depressive disorders, anti-anxiety drugs, antidepressants, primary care

## Abstract

**Background:**

Depression, anxiety and insomnia often co-occur. However, there is a lack of research regarding how they cluster and how this is related to medication used to treat them.

**Aims:**

To describe the frequencies and associations between depression, anxiety and insomnia, and treatment for these conditions in primary care.

**Method:**

A retrospective cohort study using UK electronic primary care records. We included individuals aged between 18 and 99 years old with one or more records suggesting they had a diagnosis, symptom or drug treatment for anxiety, depression or insomnia between 2015 and 2017. We report the conditional probabilities of having different combinations of diagnoses, symptoms and treatments recorded.

**Results:**

There were 1 325 960 records indicative of depression, anxiety or insomnia, for 739 834 individuals. Depression was the most common condition (*n* = 106 117 records), and SSRIs were the most commonly prescribed medication (*n* = 347 751 records). Overall, individuals with a record of anxiety were most likely to have co-occurring symptoms and diagnoses of other mental health conditions. For example, of the individuals with a record of generalised anxiety disorder (GAD), 24% also had a diagnosis of depression. In contrast, only 0.6% of those who had a diagnosis of depression had a diagnosis or symptom of GAD. Prescribing of more than one psychotropic medication within the same year was common. For example, of those who were prescribed an SNRI (serotonin-norepinephrine reuptake inhibitor), 40% were also prescribed an SSRI (selective serotonin reuptake inhibitor).

**Conclusions:**

The conditional probabilities of co-occurring anxiety, depression and insomnia symptoms, diagnoses and treatments are high.

Depression and anxiety are common mental disorders (CMD) with an increasing prevalence around the world.^1^ In 2019, the global age-standardised prevalence of a diagnosis of depression and anxiety was 3440.1 and 3779.5 per 100 000 people, respectively.^1^ However, many people experience symptoms that cannot be attributed to a single disorder and experience both anxiety and depression simultaneously (co-morbidity). The co-occurrence of these conditions results in high rates of co-morbidity compared to other psychiatric conditions.^2^

## Anxiety, depression and insomnia, and treatments

There are clear overlaps between depression and anxiety. A review found 85% of people with depression experienced symptoms of anxiety, while 90% of individuals with anxiety experienced depressive symptoms.^3^ A meta-analysis of 66 studies and 88 336 people demonstrated that all types of diagnosed anxiety predicted all types of later depressive disorders, and *vice versa*.^4^ In the Netherlands Study of Depression and Anxiety, a multi-site naturalistic cohort study (*n* = 1783), 67% of those with a depressive disorder had co-morbid anxiety, and 63% of those with anxiety also had co-morbid depression.^5^

Furthermore, insomnia has a complex relationship with mental health disorders, and there is an overlap of symptoms.^6^ Insomnia is a risk factor for, and symptom of, psychiatric conditions but can negatively impact the course and treatment of generalised anxiety disorder and depression. For example, untreated insomnia in depression is associated with depression relapse.^6^ Despite their clustering, the conditional relationships between the three conditions is not well documented.

There is an overlap in indications for, and the use of, drug treatments in clinical practice for these conditions: selective serotonin reuptake inhibitors (SSRIs) are widely used for anxiety and depression, and other antidepressants (e.g. mirtazapine) are also used in sleep disorders.^7^ Furthermore, commonly used hypnotics (benzodiazepines and Z-drugs) are associated with depression and anxiety as well as insomnia when used in the long term.^8,9^

Understanding the interrelationship between anxiety and depression is important as, when combined, the conditions are often more severe, associated with a greater suicide risk, and are more disabling, compared with each disorder alone.^10^ Their relationship to insomnia is also important as, while it is hard to determine if insomnia is primary or secondary in nature, it does lead to reduced productivity and increased healthcare costs.^11^

## Previous research in the area

There are few other studies using electronic health records (EHR) specifically exploring the frequencies of the co-occurrence of these CMDs and associated medications used to treat them. For example, a recent cohort EHR study based in the USA with 30 218 individuals found that anxiety disorders were significantly more common in treatment-resistant depression, compared with those without treatment-resistant depression (OR = 2.24; 95%CI = 2.06–2.44; *P* < 005).^12^ Evidence on the co-occurrence of CMDs and its implications is scarce, however. The aim of this study was therefore to describe the conditional probabilities of symptoms, diagnoses and treatments of depression, anxiety and insomnia, using UK primary care electronic health records; and how these conditions related to each other. We particularly focused on the associations between these conditions and medication commonly prescribed.

## Method

### Data source

The study was conceptualised in 2019. We used the IQVIA, formerly Quintiles and IMS Health, Inc Medical Research Data (IMRD), database, which includes de-identified data from The Health Improvement Network (THIN), an electronic Cegedim healthcare database containing anonymised data for 16 million registered individuals from 739 practices across the UK.^13^ The people included are broadly representative of the UK population in terms of age, gender, medical conditions and mortality.^14^ Available routine individual-level data include: demographic details, diagnoses and symptoms, laboratory tests, area level social deprivation (using the Townsend score) and prescriptions issued by GPs. Clinical events are categorised using SNOMED or Read codes, a hierarchical coding system that captures clinical information.^15^ IMRD allows long-term individual follow-up, providing longitudinal data on clinical encounters and outcomes for individual people.^16^

### UK healthcare

Healthcare in the UK is free at the point of use for all residents as part of the National Health Service (NHS). Usually, the first point of contact is in primary care, which is delivered by GPs and other healthcare professionals (nurses, pharmacists and health visitors) within a practice. During consultations, information about individuals is collected for clinical purposes. More than 98% of the population are registered with a GP, and 90% of healthcare contacts occur in primary care,^17^ which is where most CMD management occurs. While some drug treatments might be initiated in secondary care, the vast majority is continued in primary care where the prescribing budget lies.

### Study population

We included people aged between 18 and 99 who were registered with a GP for at least 1 year, from 1 April 2015 to 31 March 2017. All GP practices met standard criteria for acceptable mortality reporting (AMR) and acceptable computer usage (ACU). ACU is the date a practice was continuously entering on average at least two therapy records, one medical record and one additional health data record per patient per year;^18^ and AMR is the date a practice has comparable age, gender and generalised mortality rates with the rest of the UK.^19^

We identified individuals with one or more records entered as a SNOMED/Read code in their GP electronic health records which suggested they had a diagnosis, symptom or drug treatment based on British National Formulary (BNF) codes for anxiety, depression, insomnia or mixed anxiety and depression.

We were interested in exploring diagnoses and prescribing in recent years (2015–2017) and compiled a SNOMED/Read code and drug code list for these conditions, in a process described in previous studies.^20^ People could have more than one record of a condition/treatment but they were only counted once within the same category. If a person had a record of two different conditions/treatments in the time period, they were considered two separate events.

### Study variables

We explored different case definitions of mental health variables using data available in IMRD and Read codes to develop inclusive code lists. For a case definition of CMD, the individual had to have at least one relevant Read code recorded during the study period. Each Read code corresponded to only one of these mental health conditions, considering a code list that was validated with a rigorous mixed method approach. In this approach, two GPs constructed a list of Read codes by screening the full Read code dictionary for suitable terms. The list was then refined through consultation between six clinical academic doctors (three GPs and three psychiatrists) who agreed on relevant Read codes. We classified them into subtypes related to diagnoses, symptoms or drug treatments for depression, generalised anxiety disorder (GAD), panic disorder, other/unspecified anxiety, mixed depression and anxiety, and insomnia. The quantitative process included an exploratory factor analysis, sensitivity analysis and correspondence analysis. The qualitative classification informed by the quantitative classification found a clear clustering of codes related to depression, anxiety and insomnia. This cross-validation ensured the code list was inclusive with improved classification. The details of these methods and the results are described elsewhere.^21^

We divided prescribed medication into groups according to the BNF sub-chapters: Chapter 4.3.3 SSRI, Chapter 4.3.1 tricyclic antidepressants (TCA), Chapter 4.3.4 serotonin norepinephrine reuptake inhibitors (SNRI) and other antidepressants (e.g. mirtazapine), Chapter 4.1.2 benzodiazepines, Chapter 4.1.1 other anxiolytics (e.g. buspirone), Z-drugs and melatonin. We were interested in the prescribing of different psychotropic medication within the same year. TCA medications can be prescribed for multiple conditions, including neuropathic pain, insomnia and depression; we only included therapeutic doses for depression in our study, such as amitriptyline 50 mg or above. Medications were not included if their primary use was not for CMD; for example, propranolol was not included as its primary use is in cardiology.

### Input from people with lived experiences

A panel of people with lived experiences of anxiety and depression were involved in the interpretation of results and provided feedback on how results and conclusions were presented in this manuscript.

### Statistical analysis

We calculated the relative frequencies of any record suggestive of depression, anxiety, insomnia or mixed anxiety and depression (diagnoses and symptoms), as well as for medication prescribed to treat these conditions. We explored how these related to each other. Using cross-tables, we reported as percentages the conditional probability of having a record of a mental health condition or drug treatment given another mental health condition or treatment, and *vice versa*. In this way, the cross-table demonstrates the correlation between certain recorded mental health (MH) conditions and treatments. For example, a cell in the cross-table can report that 24% of those who had a diagnosis or symptom of GAD also had a diagnosis of depression, whereas only 0.6% of those who had a diagnosis of depression had a diagnosis or symptom of GAD. This can be described as follows: out of 100 people identified as having GAD, 24 also had depression. Conversely, out of 1000 individuals with a diagnosis of depression, only 6 people had GAD. This example is highlighted in Table 2 and Supplementary Table 1 available at https://doi.org/10.1192/bjo.2024.20.

We counted each person only once for each different combination (e.g. depression symptom and GAD, or GAD and SSRI prescription) recorded in the study period, to avoid double-counting. There was also a hierarchy in which we recorded an individual as having a diagnosis rather than a symptom if they had both a record of a diagnosis and symptom of the same condition. There was no hierarchy in relation to conditions, therefore it was possible for an individual to have, for example, a record of anxiety and depression. For variables with low frequencies, e.g. panic disorder and panic attacks, we combined diagnosis and symptom.

As our primary analysis, we examined all individuals aged 18–99 years. In further supplementary analysis, we stratified analyses by age (below 50 and 50 and above) and gender to explore differences by these factors.

### Ethical approval

The authors assert that all procedures contributing to this work comply with the ethical standards of the relevant national and institutional committees on human experimentation and with the Helsinki Declaration of 1975,^22^ as revised in 2008. Ethical approval was granted by the NHS South-East Multi-Centre Research Ethics Committee for scientific research in 2003. The IQVIA World Publications Scientific Review Committee granted scientific approval in December 2020 (SRC reference number: 20SRC057). All data were anonymised, and no individual's consent was required.

### Transparency declaration

The manuscript is an honest, accurate and transparent account of the study being reported; no important aspects of the study have been omitted.

## Results

### Characteristics

There was a total of 1 325 960 records for 739 834 individuals who were included in the study between 2015 and 2017 (Table 1) with a record indicating CMD or insomnia (a diagnosis, symptom or treatment). The mean age of individuals with a condition was between 44 (s.d. 16.1) and 47 (s.d. 16.6) years for all conditions, except for those with insomnia, where there was a higher mean age of 55 (s.d. 18.7) years. In all, 53% of individuals had a recorded diagnosis or symptom of CMD, and 47% had a recorded prescription. A total of 212 934 (29%) individuals had a recorded depression disorder or symptom, 116 594 (16%) had a recorded anxiety disorder or symptom, 22 203 (3%) had a record for the combined code ‘mixed anxiety and depression’, and 35 438 (5%) had a recorded insomnia disorder or symptom. Women were more likely to have a diagnosis or symptom of CMD (59.7–69.7%) compared with men (30.3–40.3%).

**Table 1:** Record frequencies and characteristics

Mental health diagnosis, symptom or treatment	*N* of records (%*)	Gender (%)		Age (years)	
		Women	Men	Mean	(s.d.)
Depression diagnoses	106 117 (14.3)	64.0	36.0	47.0	(16.6)
Depression symptoms	106 817 (14.4)	64.3	35.7	46.9	(17.8)
GAD	2895 (0.4)	63.2	36.8	45.8	(17.6)
Panic attack/disorder	9017 (1.2)	69.7	30.3	44.4	(17.5)
Anxiety disorder other or unspecified diagnoses	38 061 (5.1)	66.8	33.2	46.4	(17.7)
Anxiety disorder other or unspecified symptoms	66 621 (9.0)	67.7	32.3	46.9	(18.2)
Mixed depression-anxiety	22 203 (3.0)	64.5	35.5	44.0	(16.1)
Insomnia	35 438 (4.8)	59.7	40.3	55.2	(18.7)
SSRI	347 751 (47.0)	66.5	33.5	49.5	(18.8)
SNRI	110 998 (15.0)	59.8	40.2	54.1	(19.1)
TCA	180 582 (24.4)	67.1	32.9	58.0	(17.5)
Other antidepressants	10 927 (1.5)	62.1	37.9	58.6	(20.0)
Benzodiazepine	163 905 (22.2)	63.4	36.6	55.9	(19.0)
Other anxiolytic	4746 (0.6)	66.9	33.1	48.0	(17.0)
Z-drug	110 309 (14.9)	61.7	38.3	56.3	(19.3)
Melatonin	9573 (1.3)	42.8	57.2	37.7	(28.1)
*Overall (study population)*	*739 834*	*63*.*2*	*36*.*8*	*52*.*6*	*(19*.*5)*

*% = (number of records (N)/total number of people (739 834)) ×100 GAD, generalised anxiety disorder; SSRI, selective serotonin reuptake inhibitors; SNRI, serotonin norepinephrine reuptake inhibitors; TCA, tricyclic antidepressants.

Although some people were prescribed more than one medication, 347 751 (47%) individuals had a recorded SSRI prescription, 110 998 (15%) an SNRI prescription, 180 582 (24%) a TCA prescription, 163 905 (22%) a benzodiazepine prescription and 110 309 (15%) a Z drug prescription. Women were more likely to have been prescribed medication compared with men; for example, 67.1% of TCA prescriptions were for women. However, unlike the other medications, people who were prescribed melatonin were more likely to be men (57.2%) and younger, with a mean age of 38 (s.d. 28.1) years, despite it being licensed for people over 55 with insomnia. This suggests that although people were more likely to have an insomnia record if they were older, younger individuals were more likely to be prescribed melatonin. For the other medications, the mean age was between 48 (s.d. 17) and 59 (s.d. 19) years.

Frequencies of treatments, diagnoses and symptoms for depression, anxiety and insomnia can be seen in Table 2 and confidence intervals seen in Supplementary Table 1. There are no big deviations in the 95% confidence intervals presented in Supplementary Table 1; therefore, the table presented in the main text seems to provide an accurate estimate of the conditional probabilities for this population. To simplify the data, they are presented in separate tables and shown in supplementary tables, one of all symptoms and diagnoses (Supplementary Table 2), one of all treatments (Supplementary Table 3) and one for each condition: insomnia (Supplementary Table 4), anxiety (Supplementary Table 5) and depression (Supplementary Table 6).

**Table 2:** Depression, anxiety and insomnia: cross-table of treatments, diagnoses and symptoms

	Depression diagnosis	Depression symptom	GAD	Panic attack/ disorder	Anxiety disorder other or unspecified diagnosis	Anxiety disorder other or unspecified symptoms	Mixed depression-anxiety	Insomnia	SSRI	SNRI	TCA	Other antidepressants	Benzo diazepine	Other anxiolytic	Z drug	Melatonin
Depression diagnosis		29.2	0.6	1	6.6	9.6	10.4	1.2	67.7	21.7	11.8	1.6	14.5	0.9	14.4	0.5
Depression symptom	29		0.4	0.9	5.9	10.4	5.1	1.6	58.8	19.5	12.3	1.6	14.8	0.9	13.8	0.6
GAD	23.6	16		4.4	19.9	25.2	7.3	1.1	58.7	22.5	12.1	2.1	27.1	3.1	15.5	0.9
Panic attack/disorder	17.7	16.1	2.1		16.4	25.4	6.1	1.9	46.9	16.5	12.1	1.3	29.8	2.4	12.9	0.6
Anxiety disorder other or unspecified diagnosis	18.4	16.7	1.5	2.6		17.5	8	2.5	48.4	16.9	11.9	2	23.7	1.7	13.3	0.6
Anxiety disorder other or unspecified symptom	15.3	16.7	1.1	2.3	10.1		4.7	1.5	47.2	15	11.6	1.5	24.4	1.9	13.8	0.6
Mixed depression-anxiety	49.8	24.5	1	2.5	13.8	14.2		4.6	64.9	22.4	11.3	16.8	1	14.4	0.4	0.6
Insomnia	14.3	18.5	0.3	1.2	10.3	10.6	2.9		29.2	19.5	20.9	2.6	25.9	2	49.6	5
SSRI	20.7	18.1	0.5	0.8	5.3	9	4.1	0.8		12.9	13.2	1.3	17.8	0.9	14.1	0.7
SNRI	20.7	18.8	0.6	0.9	5.8	9	4.5	1.6	40.3		20.1	3.1	27.8	1.8	23.9	1.4
TCA	6.9	7.3	0.2	0.4	2.5	4.3	1.4	1.1	25.34	12.4		1.9	18.7	0.5	12.7	0.7
Other antidepressants	15.4	16	0.6	0.7	7.1	9.4	4.2	2.2	39.9	31.7	17.8		35.9	2	27.1	2.4
Benzodiazepine	9.4	9.6	0.5	1.1	5.5	9.9	2.3	1.5	37.7	18.8	20.7	2.4		1.3	19.7	1.2
Other anxiolytic	20	19.5	1.9	3	13.8	26.8	4.5	3.9	63	41	19.5	4.5	44.1		32.1	3.4
Z-drug	13.9	13.4	0.4	0.7	4.6	8.4	2.9	4.1	44.4	24	20.7	2.7	29.3	1.4		1.9
Melatonin	5.5	6.3	0.3	0.4	2.4	4.2	1	4.8	26.8	16.2	13	2.8	19.7	1.7	21.4	

GAD, generalised anxiety disorder; SSRI, selective serotonin reuptake inhibitors; SNRI, serotonin norepinephrine reuptake inhibitors; TCA, tricyclic antidepressants.

Highlighted examples highlight 24% of those who had a diagnosis or symptom of generalised anxiety disorder (GAD) also had a diagnosis of depression, whereas only 0.6% of those who had a diagnosis of depression had a diagnosis or symptom of GAD.

### Association between diagnoses and symptoms

Of the individuals who had a record of a depression diagnosis, 0.6% also had a record of GAD (highlighted in yellow in Supplementary Table 2). However, of those with a record of GAD, 24% also had a diagnosis of depression. The same was seen for panic disorder and mixed depression and anxiety. Of those people who had a depression diagnosis record, 1 and 10% also respectively had a record of panic disorder or mixed depression and anxiety (highlighted in green in Supplementary Table 2). However, 18% of those with a record of panic disorder and 50% of those with a record of mixed anxiety and depression also had a diagnosis of depression. A similar pattern was seen for insomnia: of those who had a record of a depression diagnosis, 1% also had a record of insomnia. However, of those with a record of insomnia, 14% also had a diagnosis of depression (highlighted in blue in Supplementary Table 2).

Individuals with a record of anxiety were more likely to also have records of symptoms and diagnoses of other mental health conditions, compared to depression and insomnia. Those people with GAD and panic disorder were more likely to also have a symptom of another subtype of anxiety or unspecified anxiety symptom record, compared with depression and insomnia. For example, of those people with a record of panic disorder, 25% also had a record of symptoms of unspecified anxiety. However, of those who had depressive symptoms, only 10% had a record of symptoms of unspecified anxiety disorder, and this was 2% for those with a record of insomnia (highlighted in purple in Supplementary Table 2).

### Prescribing of medications for depression, anxiety and insomnia

Prescribing of different psychotropic medication within the same year was high. SSRIs were the most likely medications to be prescribed in addition to a prescription of another medication, but were also widely used alone. For example, of those people who had an SSRI prescription, only 0.9% had a prescription of ‘other anxiolytic’ (e.g. buspirone), which is highlighted in green in Supplementary Table 3. However, of those who had a prescription of ‘other anxiolytic’, 63% also had a prescription of an SSRI (highlighted in purple in Supplementary Table 3).

‘Other anxiolytics’ and ‘other antidepressants’ were more likely to be prescribed with other medication in the same year. For example, of those people who had an ‘other anxiolytic’ prescription, 63% had a SSRI, 41% had an SNRI, 44% a benzodiazepine and 32% a Z drug (highlighted in purple in Supplementary Table 3). Of those who had an ‘other antidepressant’ prescription, 40% had a SSRI, 32% had an SNRI, 36% a benzodiazepine and 27% a Z drug (highlighted in blue in Supplementary Table 3).

### Associations according to specific conditions

Supplementary Table 4 shows (highlighted in purple) that Z drugs appeared to be most likely prescribed given an insomnia diagnosis (50%). While given an insomnia diagnosis, 29% of people were prescribed an SSRI and 5% were prescribed melatonin.

A benzodiazepine appeared most likely to be prescribed given a record of an anxiety diagnosis or symptom (highlighted in blue in Supplementary Table 5). Of those people who had panic disorder or GAD, 30 and 27% had a benzodiazepine prescription, respectively. For those who had an insomnia or depression diagnosis, this was 26 and 15%, respectively (highlighted in blue in Supplementary Tables 4 and 6). Furthermore, hypnotics (benzodiazepines and Z drugs) prescribing appeared to be high in all conditions. For example, for those with a depression diagnosis, 29% were prescribed either a benzodiazepine or Z drugs (in bold and underlined in Supplementary Table 6). For those with recorded insomnia, this was 80% (in bold and underlined in Supplementary Table 4).

Given a depression or anxiety diagnosis or symptom, an SSRI appeared most likely to be prescribed (Supplementary Table 5 and 6). For example, for those people with a depression diagnosis, 68% also had an SSRI prescription, while this was 59% for GAD, 47% for panic disorder and 48% for other/unspecified anxiety diagnoses. These examples are highlighted in yellow in Supplementary Tables 5 and 6.

TCAs appeared to be most likely prescribed in insomnia (21%). There was a similar proportion of TCA prescribing in anxiety and depression disorders and symptoms, ranging from 12.3% for symptoms of depression and 11.3% in mixed depression and anxiety. These examples are highlighted in green in Supplementary Tables 4–6.

### Differences according to age

Differences according to age can be seen in Tables 3 and 4, confidence intervals are shown in Supplementary Tables 7 and 8. In most cases the 95% confidence intervals presented in Supplementary Tables 7 and 8 are small, and there are no big deviations; therefore, the tables presented in the main text seem to provide an accurate estimate of the conditional probabilities for this population. Those people aged 50 years old or younger with CMD and insomnia were more likely prescribed an SSRI, compared with individuals over 50 years old (Tables 3 and 4). For example, in those with GAD, 63% of younger people were prescribed an SSRI, compared with 52% in those older than 50 (highlighted in purple in Tables 3 and 4). On the other hand, those people aged older than 50 with CMD appear to be more likely prescribed an SNRI or TCA, compared with younger individuals. For example, in those with GAD, 20% of individuals aged 50 or younger were prescribed an SNRI, compared to 27% in older individuals (highlighted in blue).

**Table 3:** Depression, anxiety and insomnia: relative frequency of treatments and diagnoses/symptoms, according to age (younger than 50 years old)

	Depression diagnosis	Depression symptom	GAD	Panic attack/ disorder	Anxiety disorder other or unspecified diagnosis	Anxiety disorder other or unspecified symptoms	Mixed depression-anxiety	Insomnia	SSRI	SNRI	TCA	Other antidepressants	Benzo diazepine	Other anxiolytic	Z drug	Melatonin
Depression diagnosis		31.6	0.7	1.2	7.3	10.7	11.8	1.1	69.7	20.1	9.5	1.4	13.1	1	13.5	0.4
Depression symptom	31.2		0.5	1	6.4	11.2	5.9	1.3	60.5	17.8	9.5	1.4	12.7	0.9	12.8	0.4
GAD	23.7	16.5		4.8	20.5	26.9	7.3	1.2	63	19.6	9.4	2.1	24.2	3.2	14	0.7
Panic attack/disorder	18.4	16.7	2.2		16.5	26.7	6.5	1.8	47.7	14.9	9.3	1.3	25.8	2.5	12	0.6
Anxiety disorder other or unspecified diagnosis	19.6	17.3	1.6	2.8		18	8.7	2	50.9	15.5	9.4	1.8	19.6	1.7	12	0.5
Anxiety disorder other or unspecified symptom	16.8	17.8	1.2	2.6	10.5		5.4	1.2	50	13.7	8.9	1.3	20.3	2	12.3	0.5
Mixed depression-anxiety	50.1	25.2	0.9	2.6	13.8	14.7		3.9	66.7	20.8	9.2	1.8	15.2	1	13.5	0.4
Insomnia	18.6	23.9	0.6	2	13.2	13.2	4		34.4	21.1	17.8	2.9	24.2	2.8	46.8	3.1
SSRI	23.6	20.7	0.6	1	6.4	10.8	5.3	0.7		13.1	10.4	1.1	15.7	1	13.2	0.8
SNRI	25.7	23.1	0.7	1.2	7.4	11.3	6.3	1.5	49.4		18.1	3.3	26.5	2.3	24.7	1.2
TCA	10.1	10.2	0.3	0.6	3.7	6.1	2.3	1.1	32.7	15		1.3	20.5	0.8	13.1	0.7
Other antidepressants	21.4	22.5	1	1.3	10.8	12.9	6.6	2.6	51.5	40.9	19.6		34.8	3.2	31.2	2.3
Benzodiazepine	12.2	12	0.6	1.5	6.8	12.1	3.3	1.3	43.3	19.3	18	2.1		1.8	19.6	1
Other anxiolytic	21.8	21.9	2.1	3.7	14.5	30	5.3	3.7	66.7	40.8	17.2	4.7	43.7		32.3	3.6
Z-drug	19.3	18.5	0.6	1.1	6.4	11.3	4.5	3.8	55.9	27.5	17.7	2.8	30	2		1.5
Melatonin	3.9	4.3	0.2	0.4	1.8	2.9	0.9	1.8	22.6	9.4	6.5	1.5	10.9	1.6	10.5	

GAD, generalised anxiety disorder; SSRI, selective serotonin reuptake inhibitors; SNRI, serotonin norepinephrine reuptake inhibitors; TCA, tricyclic antidepressants.

Purple highlighted example shows of the younger people with GAD, 63% were prescribed an SSRI. Blue highlighted example shows of the younger people with GAD, 20% were prescribed an SNRI. Green highlighted example shows of the younger people with panic disorder, 26% were prescribed benzodiazepines. Yellow highlighted examples show of the younger people with symptoms of unspecified anxiety, 20% had a benzodiazepine prescription.

**Table 4:** Depression, anxiety and insomnia: relative frequency of treatments and diagnoses/symptoms, according to age (older than 50 years old)

	Depression diagnosis	Depression symptom	GAD	Panic attack/ disorder	Anxiety disorder other or unspecified diagnosis	Anxiety disorder other or unspecified symptoms	Mixed depression-anxiety	Insomnia	SSRI	SNRI	TCA	Other antidepressants	Benzo diazepine	Other anxiolytic	Z drug	Melatonin
Depression diagnosis		25.7	0.6	0.8	5.6	8	8.5	1.5	64.7	24	15.1	1.9	16.6	0.8	15.7	0.7
Depression symptom	25.8		0.4	0.7	5.3	9.1	3.9	1.9	56.4	22.1	16.5	2	17.9	0.8	15.4	0.8
GAD	23.4	15.1		3.6	18.8	22.4	7.3	0.7	51.7	27.1	16.5	2.2	31.9	2.9	17.8	1.3
Panic attack/disorder	16.3	14.9	2		16.3	23	5.4	1.9	45.4	19.6	17.6	1.5	37.9	2.1	14.8	0.6
Anxiety disorder other or unspecified diagnosis	16.6	15.7	1.4	2.2		16.6	6.9	3.3	44.5	19.2	15.8	2.3	30.1	1.8	15.5	0.8
Anxiety disorder other or unspecified symptom	13.1	15	0.9	1.8	9.2		3.7	1.9	43	17	15.7	1.9	30.6	1.7	16.1	0.8
Mixed depression-anxiety	49.4	22.9	1.1	2.2	13.6	13.2		5.9	61.2	25.4	15.6	2.6	20.4	0.9	16.3	0.5
Insomnia	11.5	15	0.1	0.7	8.5	8.9	2.1		25.8	18.5	22.9	2.4	27	1.5	51.4	6.3
SSRI	17.3	15	0.4	0.6	4	7	2.8	0.9		12.6	16.3	1.4	20.1	0.7	15.1	0.7
SNRI	16.8	15.4	0.5	0.6	4.6	7.3	3	1.7	33		21.7	3	28.8	1.3	23.3	1.6
TCA	5.4	5.9	0.2	0.3	1.9	3.4	1	1.1	21.8	11		1	17.9	0.4	12.4	0.7
Other antidepressants	12	12.2	0.4	0.4	4.9	7.3	2.8	1.9	33.3	26.5	16.8		36.5	1.2	24.7	2.5
Benzodiazepine	7.5	8	0.4	0.8	4.6	8.4	1.7	1.6	33.8	18.5	22.5	2.6		0.9	19.8	1.3
Other anxiolytic	17.5	16.3	1.6	2.1	12.9	22.5	3.4	4.1	58.1	41.3	22.7	4.2	44.8		31.9	3.2
Z-drug	10.3	10	0.3	0.5	3.4	6.4	1.8	4.3	36.8	21.7	22.7	2.6	28.8	1		2.1
Melatonin	8.6	9.8	0.4	0.4	3.5	6.5	1.1	10.3	34.5	28.8	24.7	5.1	35.9	1.9	41.2	

GAD, generalised anxiety disorder; SSRI, selective serotonin reuptake inhibitors; SNRI, serotonin norepinephrine reuptake inhibitors; TCA, tricyclic antidepressants.

Purple highlighted example shows of the older people with GAD, 52% were prescribed an SSRI.

Blue highlighted example shows of the older people with GAD, 27% were prescribed an SNRI. Green highlighted example shows of the older people with panic disorder, 38% were prescribed a benzodiazepine. Yellow highlighted examples show of the older people with symptoms of unspecified anxiety, 30-31% had a benzodiazepine prescription.

Benzodiazepines were more commonly prescribed for older individuals; for older people with panic disorder, 38% were prescribed a benzodiazepine, compared with 26% of younger people with the same condition (highlighted in green). However, benzodiazepines were not the most commonly prescribed medication in older people with a record of insomnia, but they were frequently issued for those with a record of ‘other/ unspecified anxiety’ symptoms and diagnoses, and panic disorder. In those people aged 50 or younger with symptoms of unspecified anxiety, 20% had a benzodiazepine prescription, compared with 30–31% in those older than 50 (highlighted in yellow).

### Differences according to gender

There were no big differences seen between men and women. Given a diagnosis or symptom of depression, anxiety or insomnia, there appear to be no meaningful gender differences in the probability of receiving treatment with psychotropic medication, and also the co-occurrence of CMD.

## Discussion

### Summary

This is the first study outlining the associations between GP diagnostic and symptom recording of depression, anxiety and insomnia, and drug prescribing for these conditions over a two-year period. People with a record of anxiety appeared more likely to also have symptoms/diagnoses of other anxieties, depression or insomnia, compared with individuals with a record of depression or insomnia. People with depression were more likely to have a sole diagnosis or symptom of depression and not have co-occurring mental health conditions. Prescribing of different medications within the same year was common, especially if there was a record of ‘other anxiolytic’ e.g. buspirone and ‘other antidepressant’ e.g. mirtazapine, perhaps reflecting that these might commonly be second-line treatments following treatment with an SSRI. Finally, there was high prescribing of hypnotics across all conditions.

### Comparison with existing literature

Anxiety appeared more likely to co-exist with other mental health conditions, compared with depression and insomnia. This is in contrast to a previous meta-analysis which found a bi-directional relationship of equal measure between anxiety and depression,^4^ although not all included studies represented the general population, and some were in specialised settings. Symptoms of anxiety and depression overlap but form separate diagnoses in the International Classification of Diseases/Diagnostic and Statistical Manual of Mental Disorders (ICD/DSM). However, compared with depression, anxiety can be underdiagnosed or misdiagnosed and therefore remains untreated, especially in older people where it can be viewed by healthcare professionals as normal.^23,24^ Our results could therefore be due to anxiety being under-recognised and unaddressed in some individuals with depression, resulting in additional or worsening depression and anxiety. If these people do not receive appropriate treatment, it can result in frequent use of both primary and secondary care services.^25^

We also found the frequency of prescribing for some medication to be high, in particular SSRIs in people taking other medications. Individuals may be prescribed one class of drug following side effects or the treatment failure of another. It is known that access to psychological therapies is variable (e.g. for those people with long-term conditions) with potentially long waiting times.^26^ The use of different drug classes by the same individual may reflect unmet need in the management of anxiety and depression and a lack of access to recommended non-pharmacological treatments. Improved access to psychological therapies could reduce the need for prescribed medication. This could reduce harmful side-effects and interactions between medications, which is especially relevant to older people.^27^ However, psychological treatments are not well recorded in primary care, and it is possible that individuals may receive therapy as well as medication.

Hypnotic prescribing was high in all conditions, and especially benzodiazepine prescribing in GAD and panic disorder, despite their well-known harmful side-effects.^7,8^ Although it is recommended that benzodiazepines should be used for short-term use, our analyses cannot tell whether they are used for long-term use. Due to time constraints, GPs may feel there is a lack of alternatives and are overwhelmed by the psychosocial problems of their patients.^28^ There are around 300 000 long-term benzodiazepine users in the UK,^29^ and high prescribing rates were associated with increased misuse.^30^ Although benzodiazepines are effective in short-term use, long-term users are recommended to slowly reduce benzodiazepine use over time, but this can take over a year.^31^ This could explain why there is prescribing of more than one class of medication, as doctors try to de-prescribe benzodiazepines, replacing it with alternatives.

Older people with CMD appeared to be less commonly prescribed SSRIs and more commonly prescribed SNRIs and TCAs, compared with those under 50 years, although the National Institute for Health and Care Excellence (NICE) recommend the SSRIs should be the first line.^32^ This could reflect treatment failure of SSRIs and reflect prescribing patterns in the UK, where clinicians may be more aware of certain SSRI side-effects in the elderly, e.g. hyponatraemia, while SNRIs may be regarded to be safer.^33^

It was surprising to find TCA prescribing to be higher in older people, given that TCAs are not considered as safe as SSRIs and SNRIs, due to side-effects including sedation and orthostatic hypotension.^34^ TCAs can be prescribed for multiple symptoms, such as depression, insomnia and neurological pain and may be more favoured in older adults, as shown by a cohort study of primary care data that included 60 746 people aged 65 years and older with depression, which compared outcomes of SSRI, TCA, MAOI and other antidepressants. They found a similar side-effect profile for all drugs.^35^ Our results may reflect legacy prescriptions, where medication should be discontinued after a certain period. Legacy pharmacy is prevalent, accounting for 46% of people taking antidepressants in a primary care study (3766 out of 8119 people).^36^

### Strengths and limitations

To the best of our knowledge, this is the largest population-based study to date describing the association between different common mental disorders, insomnia and the probability of drug prescribing for these conditions. We used a rigorous cross-validation process to define each condition, with input from six clinicians (GPs and psychiatrists). These included a wide range of diagnoses, symptoms and treatment codes as markers to fully capture these conditions. This is a broader approach than previous research, and we believe it better reflects how common mental disorders are recorded in primary care. For example, an individual may be prescribed an SSRI and have symptoms of depression recorded but not have a depression diagnosis. We stratified by broad age bands for this exploratory analysis, and further work could explore in more detail if there are differences by narrower age categories.

Our results are also limited by what information has been entered in the medical records by healthcare professionals. It is likely that sometimes common mental disorders are recognised and discussed in consultations but not recorded, due to issues such as stigma or wanting to avoid the use of labels of mental disorder, e.g. where the problem is felt to be largely social in origin or to avoid additional workload by giving individuals mental health diagnoses.^37^ We used a national sample from the UK, and findings may not be applicable outside the UK with different healthcare systems.

We used just one record of a prescription to indicate a mental health condition, which does not guarantee that the medication prescribed has been collected from the pharmacy and used; however, it is likely to reflect an underlying mental health condition that triggered the initiation of the medication. It may be that the decision to prescribe medication drives the recording of symptoms and diagnoses, and that those where no treatment is prescribed are more likely to go undiagnosed in their healthcare records. Another limitation is that medications may be prescribed for other conditions not captured in this study, for example, benzodiazepines which are prescribed in other conditions such as epilepsy, leading to an overestimation of some of the figures. Moreover, we excluded a few medications that are often prescribed for other reasons, e.g. low doses of amitriptyline which are prescribed for depression, insomnia and pain. Finally, it was not possible to measure the appropriateness of the drug prescribing with the data that was available; to answer this, a different study design would have been required.

### Implications for practice and research

Co-occurrence of different common mental health conditions and insomnia is high, particularly for those people with recorded anxiety diagnoses and symptoms. This is important, as there is far less research conducted in anxiety compared to depression,^38^ and future research should therefore focus on why anxiety is more likely to be co-morbid compared with depression. Given a recorded diagnosis or symptom, the probability of a prescription of a related drug treatment, e.g. an antidepressant, anxiolytic or hypnotic, is very high (up to 67%), and prescribing of different psychotropic medications within the same time period is widespread. This highlights the burden of these conditions for individuals that present to primary care and have their condition recognised and recorded. Further research by our team will include investigating interrelationships with other mental health conditions and medications, such as severe mental illness and antipsychotics. Beyond that, future research could investigate how associations of co-occurring mental health conditions and prescriptions relate to outcomes. It would be useful to identify if resources, services or treatment options need to be tailored or developed to manage the large number of people with co-occurring mental health conditions. In addition, future research could investigate the appropriateness of prescribing for CMD in primary care, explore reasons for deviation from treatment guidelines and explore the balance between risks and benefits when these deviations occur. We explored the years 2015–17, before the COVID-19 pandemic, and future research could compare co-occurring CMD from 2020 and differences since the pandemic. Finally, to understand if there are meaningful differences according to demographics, such as age, tests for interactions could be conducted.

In conclusion, there is a high proportion of recorded co-occurring anxiety, depression and insomnia symptoms and diagnoses in primary care. Given a recorded diagnosis or symptom of depression, anxiety or insomnia in primary care, the probability of a related drug treatment is high, with prescribing of multiple psychotropic medications in the same year being common. This highlights the high burden of these conditions for those people who have common mental disorders identified and recorded in primary care.

## Supporting information

Nimmons et al. supplementary material Nimmons et al. supplementary material

## Data Availability

The data that support the findings of this study are openly available in Zenodo at https://doi.org/10.5281/zenodo.10535386.
