# Supplementary material for: Exploring the co-occurrence of depression, anxiety and insomnia symptoms, diagnoses and treatments in primary care: observational study using UK primary care data
Source: BJPsych Open. 2024 Apr 18;10(3):e76. doi: 10.1192/bjo.2024.20 (PMC11060065; doi:10.1192/bjo.2024.20)
Supplement: Nimmons et al. supplementary material [file S2056472424000206sup001.docx]

**Supplementary Tables**

| Depression-diagnosis |  | 29.2(28.9-29.5) | 0.6(0.6-0.7) | 1.0(0.9-1.1) | 6.6(6.5-6.8) | 9.6(9.4-9.8) | 10.4(10.0-11.0) | 1.2(1.2-1.3) | 67.7(67.4-68.0) | 21.7(21.4-21.9) | 11.8(11.6-12.0) | 1.6(1.5-1.7) | 14.5(14.3-14.7) | 0.9(0.8-1.0) | 14.4(14.2-14.6) | 0.5(0.5-0.5) |
| --- | --- | --- | --- | --- | --- | --- | --- | --- | --- | --- | --- | --- | --- | --- | --- | --- |
| Depression-symptom | 29 (28.7-29.3) |  | 0.4(0.4-0.5) | 0.9(0.8-1.0) | 5.9(5.8-6.1) | 10.4(10.2-10.6) | 5.1(4.4-6.0) | 1.6(1.5-1.7) | 58.8(58.5-59.1) | 19.5(19.3-19.8) | 12.3(12.1-12.5) | 1.6(1.6-1.7) | 14.8(14.6-15.0) | 0.9(0.8-0.9) | 13.8(13.6-14.0) | 0.6(0.5-0.6) |
| Generalised AD | 23.6(22.1-25.2) | 16.0(14.6-17.3) |  | 4.4(3.6-5.2) | 19.9(18.4-21.4) | 25.2(23.6-26.8) | 7.3(6.4-7.9) | 1.1(0.7-1.5) | 58.7(56.8-60.5) | 22.5(21.0-24.0) | 12.1(11.0-13.4) | 2.1(1.6-2.7) | 27.1(25.5-28.7) | 3.1(2.5-3.8) | 15.5(14.2-16.8) | 0.9(0.6-1.3) |
| Panic attack/disorder | 17.7(16.7-18.7) | 16.1(15.2-17.0) | 2.1(1.8-2.5) |  | 16.4(15.5-17.4) | 25.4(24.3-26.5) | 6.1(5.6-6.6) | 1.9(1.6-2.3) | 46.9(45.6-48.2) | 16.5(15.6-17.5) | 12.1(11.3-12.9) | 1.3(1.1-1.7) | 29.8(28.7-31.0) | 2.4(2.0-2.8) | 12.9(12.1-13.8) | 0.6(0.4-0.8) |
| Anxiety Disorder other or unspecified-diagnosis | 18.4(18.0-18.8) | 16.7(16.3-17.1) | 1.5(1.4-1.6) | 2.6(2.4-2.7) |  | 17.5(17.1-17.8) | 8.0(7.5-8.4) | 2.5(2.3-2.7) | 48.4(47.9-48.9) | 16.9(16.5-17.3) | 11.9(11.6-12.2) | 2.0(1.9-2.2) | 23.7(23.2-24.1) | 1.7(1.6-1.9) | 13.3(13.0-13.7) | 0.6(0.5-0.7) |
| Anxiety Disorder other or unspecified-symptom | 15.3(15.1-15.6) | 16.7(16.4-17.0) | 1.1(1.0-1.2) | 2.3(2.2-2.4) | 10.1(9.8-10.2) |  | 4.7(4.2-5.2) | 1.5(1.4-1.6) | 47.2(46.8-47.6) | 15.0(14.8-15.3) | 11.6(11.3-11.8) | 1.5(1.4-1.6) | 24.4(24.1-24.7) | 1.9(1.8-2.0) | 13.8(13.6-14.1) | 0.6(0.5-0.7) |
| Mixed Depression-Anxiety | 49.8(49.1-50.1) | 24.5(23.9-25.0) | 1.0(1.0-1.1) | 2.5(2.2-2.7) | 13.8(13.3-14.3) | 14.2(13.8-15.0) |  | 4.6(4.1-5.0) | 64.9(63.8-65.5) | 22.4(21.7-23.0) | 11.3(10.7-11.7) | 16.8(16.2-17.2) | 1.0(0.9-1.2) | 14.4(13.9-14.9) | 0.4(0.3-0.6) | 0.6(0.4-0.7) |
| Insomnia | 14.3(13.6-15.0) | 18.5(17.7-19.3) | 0.3(0.2-0.5) | 1.2(1.0-1.45) | 10.3(9.7-11.0) | 10.6(10.0-11.2) | 2.9(2.5-3.4) |  | 29.2(28.3-30.1) | 19.5(18.7-20.3) | 20.9(20.1-21.8) | 2.6(2.2-2.9) | 25.9(25.0-26.8) | 2.0(1.7-2.3) | 49.6(48.6-50.7) | 5.0(5.0-5.5) |
| SSRI | 20.7(20.5-20.8) | 18.1(17.9-18.2) | 0.5(0.5-0.5) | 0.8(0.8-0.8) | 5.3(5.2-5.4) | 9.0(8.9-9.1) | 4.1(3.7-4.6) | 0.8(0.7-0.8) |  | 12.9(12.7-13.0) | 13.2(13.1-13.3) | 1.3(1.2-1.3) | 17.8(17.6-17.9) | 0.9(0.8-0.9) | 14.1(13.9-14.2) | 0.7(0.7-0.8) |
| SNRI | 20.7(20.5-21.0) | 18.8(18.6-19.0) | 0.6(0.5-0.6) | 0.9(0.8-1.0) | 5.8(5.7-5.9) | 9.0(8.9-9.2) | 4.5(4.0-5.1) | 1.6(1.5-1.7) | 40.3(40.0-40.6) |  | 20.1(19.9-20.3) | 3.1(3.0-3.2) | 27.8(27.5-28.1) | 1.8(1.7-1.8) | 23.9(23.6-24.1) | 1.4(1.3-1.5) |
| TCA | 6.9(6.8-7.0) | 7.3(7.2-7.4) | 0.2(0.2-0.2) | 0.4(0.4-0.4) | 2.5(2.4-2.6) | 4.3(4.2-4.4) | 1.4(.9-1.9) | 1.1(1.0-1.1) | 25.34(25.2-25.6) | 12.4(12.2-12.5) |  | 1.9(1.0-1.1) | 18.7(18.6-18.9) | 0.5(0.5-0.6) | 12.7(12.5-12.8) | 0.7(0.7-0.7) |
| Other antidepressants | 15.4(14.7-16.1) | 16.0(15.3-16.7) | 0.6(0.4-0.7) | 0.7(0.6-0.9) | 7.1(6.6-7.6) | 9.4(8.8-9.9) | 4.2(3.8-4.8) | 2.2(1.9-2.5) | 39.9(39.0-40.8) | 31.7(30.9-32.6) | 17.8(17.1-18.5) |  | 35.9(34.9-36.7) | 2.0(1.7-2.2) | 27.1(26.2-27.9) | 2.4(2.1-2.7) |
| Benzodiazepine | 9.4(9.3-9.6) | 9.6(9.5-9.8) | 0.5(0.5-0.5) | 1.1(1.0-1.1) | 5.5(5.4-5.6) | 9.9(9.8-10.1) | 2.3(1.6-2.7) | 1.5(1.4-1.5) | 37.7(37.5-37.9) | 18.8(18.6-19.0) | 20.7(20.5-20.9) | 2.4(2.3-2.5) |  | 1.3(1.2-1.3) | 19.7(19.5-19.9) | 1.2(1.1-1.2) |
| Other anxiolytic | 20.0(18.8-21.1) | 19.5(18.4-20.7) | 1.9(1.5-2.3) | 3.0(2.5-3.5) | 13.8(12.9-14.8) | 26.8(25.5-28.0) | 4.5(4.0-4.9) | 3.9(3.4-4.5) | 63.0(61.7-64.4) | 41.0(40.0-42.0) | 19.5(18.4-20.7) | 4.5(3.9-5.1) | 44.1(42.7-45.6) |  | 32.1(30.7-33.4) | 3.4(2.9-3.9) |
| Z-drug | 13.9(13.7-14.1) | 13.4(13.2-13.6) | 0.4(0.4-0.5) | 0.7(0.7-0.8) | 4.6(4.5-4.7) | 8.4(8.2-8.5) | 2.9(2.45-3.5) | 4.1(4.0-4.2) | 44.4(44.1-44.7) | 24.0(23.8-24.3) | 20.7(20.5-20.9) | 2.7(2.6-2.8) | 29.3(29.0-29.5) | 1.4(1.3-1.5) |  | 1.9(1.8-1.9) |
| Melatonin | 5.5(5.1-6.0) | 6.3(5.8-6.8) | 0.3(0.2-0.4) | 0.4(0.3-0.5) | 2.4(2.1-2.7) | 4.2(3.8-4.6) | 1.0(0.9-1.2) | 4.8(4.4-5.3) | 26.8(25.9-27.7) | 16.2(15.5-17.0) | 13.0(12.3-13.6) | 2.8(2.4-3.1) | 19.7(18.9-20.5) | 1.7(1.4-1.9) | 21.4(20.5-22.2) |  |
|  | Depression diagnosis | Depression symptom | Generalised AD | Panic attack/ disorder | Anxiety disorder other or unspecified diagnosis | Anxiety disorder other or unspecified symptoms | Mixed Depression Anxiety | Insomnia | SSRI | SNRI | TCA | Other antidepressants | Benzo diazepine | Other anxiolytic | Z drug | Melatonin |

Supplementary Table 1: Depression, anxiety and insomnia: cross-table of treatments, diagnoses and symptoms with confidence intervals

| Depression-diagnosis |  | 29.2 | 0.6 | 1 | 6.6 | 9.6 | 10.4 | 1.2 |
| --- | --- | --- | --- | --- | --- | --- | --- | --- |
| Depression-symptom | 29 |  | 0.4 | 0.9 | 5.9 | 10.4 | 5.1 | 1.6 |
| Generalised AD | 23.6 | 16 |  | 4.4 | 19.9 | 25.2 | 7.3 | 1.1 |
| Panic attack/disorder | 17.7 | 16.1 | 2.1 |  | 16.4 | 25.4 | 6.1 | 1.9 |
| Anxiety Disorder other or unspecified-diagnosis | 18.4 | 16.7 | 1.5 | 2.6 |  | 17.5 | 8 | 2.5 |
| Anxiety Disorder other or unspecified-symptom | 15.3 | 16.7 | 1.1 | 2.3 | 10.1 |  | 4.7 | 1.5 |
| Mixed Depression-Anxiety | 49.8 | 24.5 | 1 | 2.5 | 13.8 | 14.2 |  | 4.6 |
| Insomnia | 14.3 | 18.5 | 0.3 | 1.2 | 10.3 | 10.6 | 2.9 |  |
|  | Depression diagnosis | Depression symptom | Generalised AD | Panic attack/ disorder | Anxiety disorder other or unspecified diagnosis | Anxiety disorder other or unspecified symptoms | Mixed Depression Anxiety | Insomnia |

Supplementary Table 2: Diagnoses and symptoms for depression, anxiety and insomnia

| SSRI |  | 12.9 | 13.2 | 1.3 | 17.8 | 0.9 | 14.1 | 0.7 |
| --- | --- | --- | --- | --- | --- | --- | --- | --- |
| SNRI | 40.3 |  | 20.1 | 3.1 | 27.8 | 1.8 | 23.9 | 1.4 |
| TCA | 25.34 | 12.4 |  | 1.9 | 18.7 | 0.5 | 12.7 | 0.7 |
| Other antidepressants | 39.9 | 31.7 | 17.8 |  | 35.9 | 2 | 27.1 | 2.4 |
| Benzodiazepine | 37.7 | 18.8 | 20.7 | 2.4 |  | 1.3 | 19.7 | 1.2 |
| Other anxiolytic | 63 | 41 | 19.5 | 4.5 | 44.1 |  | 32.1 | 3.4 |
| Z-drug | 44.4 | 24 | 20.7 | 2.7 | 29.3 | 1.4 |  | 1.9 |
| Melatonin | 26.8 | 16.2 | 13 | 2.8 | 19.7 | 1.7 | 21.4 |  |
|  | SSRI | SNRI | TCA | Other antidepressants | Benzo diazepine | Other anxiolytic | Z drug | Melatonin |

Supplementary Table 3: Medication prescribing for depression, anxiety and insomnia

| Insomnia |  | 29.2 | 19.5 | 20.9 | 2.6 | **25.9** | 2 | **49.6** | 5 |
| --- | --- | --- | --- | --- | --- | --- | --- | --- | --- |
| SSRI | 0.8 |  | 12.9 | 13.2 | 1.3 | 17.8 | 0.9 | 14.1 | 0.7 |
| SNRI | 1.6 | 40.3 |  | 20.1 | 3.1 | 27.8 | 1.8 | 23.9 | 1.4 |
| TCA | 1.1 | 25.34 | 12.4 |  | 1.9 | 18.7 | 0.5 | 12.7 | 0.7 |
| Other antidepressants | 2.2 | 39.9 | 31.7 | 17.8 |  | 35.9 | 2 | 27.1 | 2.4 |
| Benzodiazepine | 1.5 | 37.7 | 18.8 | 20.7 | 2.4 |  | 1.3 | 19.7 | 1.2 |
| Other anxiolytic | 3.9 | 63 | 41 | 19.5 | 4.5 | 44.1 |  | 32.1 | 3.4 |
| Z-drug | 4.1 | 44.4 | 24 | 20.7 | 2.7 | 29.3 | 1.4 |  | 1.9 |
| Melatonin | 4.8 | 26.8 | 16.2 | 13 | 2.8 | 19.7 | 1.7 | 21.4 |  |
|  | Insomnia | SSRI | SNRI | TCA | Other antidepressants | Benzo diazepine | Other anxiolytic | Z drug | Melatonin |

Supplementary Table 4: Associations according to specific conditions: Insomnia treatments and combined symptoms and diagnoses

| Generalised AD |  | 4.4 | 19.9 | 25.2 | 7.3 | 58.7 | 22.5 | 12.1 | 2.1 | 27.1 | 3.1 | 15.5 | 0.9 |
| --- | --- | --- | --- | --- | --- | --- | --- | --- | --- | --- | --- | --- | --- |
| Panic attack/disorder | 2.1 |  | 16.4 | 25.4 | 6.1 | 46.9 | 16.5 | 12.1 | 1.3 | 29.8 | 2.4 | 12.9 | 0.6 |
| Anxiety Disorder other or unspecified-diagnosis | 1.5 | 2.6 |  | 17.5 | 8 | 48.4 | 16.9 | 11.9 | 2 | 23.7 | 1.7 | 13.3 | 0.6 |
| Anxiety Disorder other or unspecified-symptom | 1.1 | 2.3 | 10.1 |  | 4.7 | 47.2 | 15 | 11.6 | 1.5 | 24.4 | 1.9 | 13.8 | 0.6 |
| Mixed Depression-Anxiety | 1 | 2.5 | 13.8 | 14.2 |  | 64.9 | 22.4 | 11.3 | 16.8 | 1 | 14.4 | 0.4 | 0.6 |
| SSRI | 0.5 | 0.8 | 5.3 | 9 | 4.1 |  | 12.9 | 13.2 | 1.3 | 17.8 | 0.9 | 14.1 | 0.7 |
| SNRI | 0.6 | 0.9 | 5.8 | 9 | 4.5 | 40.3 |  | 20.1 | 3.1 | 27.8 | 1.8 | 23.9 | 1.4 |
| TCA | 0.2 | 0.4 | 2.5 | 4.3 | 1.4 | 25.34 | 12.4 |  | 1.9 | 18.7 | 0.5 | 12.7 | 0.7 |
| Other antidepressants | 0.6 | 0.7 | 7.1 | 9.4 | 4.2 | 39.9 | 31.7 | 17.8 |  | 35.9 | 2 | 27.1 | 2.4 |
| Benzodiazepine | 0.5 | 1.1 | 5.5 | 9.9 | 2.3 | 37.7 | 18.8 | 20.7 | 2.4 |  | 1.3 | 19.7 | 1.2 |
| Other anxiolytic | 1.9 | 3 | 13.8 | 26.8 | 4.5 | 63 | 41 | 19.5 | 4.5 | 44.1 |  | 32.1 | 3.4 |
| Z-drug | 0.4 | 0.7 | 4.6 | 8.4 | 2.9 | 44.4 | 24 | 20.7 | 2.7 | 29.3 | 1.4 |  | 1.9 |
| Melatonin | 0.3 | 0.4 | 2.4 | 4.2 | 1 | 26.8 | 16.2 | 13 | 2.8 | 19.7 | 1.7 | 21.4 |  |
|  | Generalised AD | Panic attack/ disorder | Anxiety disorder other or unspecified diagnosis | Anxiety disorder other or unspecified symptoms | Mixed Depression Anxiety | SSRI | SNRI | TCA | Other antidepressants | Benzo diazepine | Other anxiolytic | Z drug | Melatonin |

Supplementary Table 5: Associations according to specific conditions: Anxiety treatments, diagnoses and symptoms

| Depression-diagnosis |  | 29.2 | 10.4 | 67.7 | 21.7 | 11.8 | 1.6 | **14.5** | 0.9 | **14.4** | 0.5 |
| --- | --- | --- | --- | --- | --- | --- | --- | --- | --- | --- | --- |
| Depression-symptom | 29 |  | 5.1 | 58.8 | 19.5 | 12.3 | 1.6 | 14.8 | 0.9 | 13.8 | 0.6 |
| Mixed Depression-Anxiety | 49.8 | 24.5 |  | 64.9 | 22.4 | 11.3 | 16.8 | 1 | 14.4 | 0.4 | 0.6 |
| SSRI | 20.7 | 18.1 | 4.1 |  | 12.9 | 13.2 | 1.3 | 17.8 | 0.9 | 14.1 | 0.7 |
| SNRI | 20.7 | 18.8 | 4.5 | 40.3 |  | 20.1 | 3.1 | 27.8 | 1.8 | 23.9 | 1.4 |
| TCA | 6.9 | 7.3 | 1.4 | 25.34 | 12.4 |  | 1.9 | 18.7 | 0.5 | 12.7 | 0.7 |
| Other antidepressants | 15.4 | 16 | 4.2 | 39.9 | 31.7 | 17.8 |  | 35.9 | 2 | 27.1 | 2.4 |
| Benzodiazepine | 9.4 | 9.6 | 2.3 | 37.7 | 18.8 | 20.7 | 2.4 |  | 1.3 | 19.7 | 1.2 |
| Other anxiolytic | 20 | 19.5 | 4.5 | 63 | 41 | 19.5 | 4.5 | 44.1 |  | 32.1 | 3.4 |
| Z-drug | 13.9 | 13.4 | 2.9 | 44.4 | 24 | 20.7 | 2.7 | 29.3 | 1.4 |  | 1.9 |
| Melatonin | 5.5 | 6.3 | 1 | 26.8 | 16.2 | 13 | 2.8 | 19.7 | 1.7 | 21.4 |  |
|  | Depression diagnosis | Depression symptom | Mixed Depression Anxiety | SSRI | SNRI | TCA | Other antidepressants | Benzo diazepine | Other anxiolytic | Z drug | Melatonin |

Supplementary Table 6: Associations according to specific conditions: Depression treatments, diagnoses and symptoms

| Depression-diagnosis |  | 31.6 (31.2-31.9) | 0.7 (0.6-0.7) | 1.2 (1.1-1.3) | 7.3 (7.1-7.5) | 10.7 (10.5-11.0) | 11.8 (11.3-12.4) | 1.1 (1.0-1.1) | 69.7 (69.4-70.1) | 20.1 (19.8-20.4) | 9.5 (9.3-9.7) | 1.4 (1.3-1.4) | 13.1 (12.8-13.3) | 1.0 (0.9-1.0) | 13.5 (13.3-13.8) | 0.4 (0.3-0.4) |
| --- | --- | --- | --- | --- | --- | --- | --- | --- | --- | --- | --- | --- | --- | --- | --- | --- |
| Depression-symptom | 31.2 (30.8-31.6) |  | 0.5 (0.4-0.5) | 1.0 (1.0-1.1) | 6.4 (6.2-6.6) | 11.2 (11-11.5) | 5.9 (5.3-6.4) | 1.3 (1.3-1.4) | 60.5 (60.1-60.8) | 17.8 (17.5-18.1) | 9.5 (9.3-9.7) | 1.4 (1.3-1.5) | 12.7 (12.4-13.0) | 0.9 (0.9-1.0) | 12.8 (12.5-13.0) | 0.4 (0.4-0.5) |
| Generalised AD | 23.7 (21.7-25.7) | 16.5 (14.8-18.3) |  | 4.8 (3.9-5.9) | 20.5 (18.7-22.5) | 26.9 (24.9-29.1) | 7.3 (6.7-7.8) | 1.2 (0.8-1.9) | 63.0 (60.7-65.2) | 19.6 (17.8-21.5) | 9.4 (8.1-10.9) | 2.1 (1.5-2.9) | 24.2 (22.2-26.2) | 3.2 (2.4-4.1) | 14.0 (12.5-15.7) | 0.7 (0.4-1.2) |
| Panic attack/disorder | 18.4 (17.2-19.6) | 16.7 (15.5-17.9) | 2.2 (1.7-2.7) |  | 16.5 (15.4-17.7) | 26.7 (25.3-28.1) | 6.5 (6.0-7.0) | 1.8 (1.5-2.3) | 47.7 (46.1-49.3) | 14.9 (13.9-16.1) | 9.3 (8.4-10.3) | 1.3 (0.9-1.7) | 25.8 (24.4-27.2) | 2.5 (2.1-3.1) | 12.0 (11.0-13.0) | 0.6 (0.4-0.9) |
| Anxiety Disorder other or unspecified-diagnosis | 19.6 (19.1-20.1) | 17.3 (16.8-17.8) | 1.6 (1.4-1.7) | 2.8 (2.6-3.0) |  | 18 (17.5-18.5) | 8.7 (8.2-9.0) | 2.0 (1.8-2.2) | 50.9 (50.2-51.5) | 15.5 (15-15.9) | 9.4 (9.1-9.8) | 1.8 (1.7-2.0) | 19.6 (19.1-20.1) | 1.7 (1.5-1.9) | 12.0 (11.6-12.4) | 0.5 (0.4-0.6) |
| Anxiety Disorder other or unspecified-symptom | 16.8 (16.4-17.2) | 17.8 (17.4-18.2) | 1.2 (1.1-1.3) | 2.6 (2.5-2.8) | 10.5 (10.2-10.8) |  | 5.4 (4.9-6.0) | 1.2 (1.1-1.3) | 50.0 (49.5-50.5) | 13.7 (13.4-14.1) | 8.9 (8.7-9.2) | 1.3 (1.2-1.4) | 20.3 (19.9-20.7) | 2.0 (1.9-2.2) | 12.3 (12.0-12.7) | 0.5 (0.4-0.5) |
| Mixed Depression-Anxiety | 50.1 (49.7-52.5) | 25.2 (24.1-25.9) | 0.9 (0.8-1.1) | 2.6 (2.0-3.0) | 13.8 (12.5-14.2) | 14.7 (14.2-15.5) |  | 3.9 (3.2-4.5) | 66.7 (66.1-67.5) | 20.8 (20.1-21.6) | 9.2 (8.7-9.9) | 1.8 (1.4-2.1) | 15.2 (14.6-15.8) | 1.0 (0.9-1.2) | 13.5 (13.0-14.0) | 0.4 (0.1-0.8) |
| Insomnia | 18.6 (17.3-19.9) | 23.9 (22.5-25.3) | 0.6 (0.4-1.0) | 2.0 (1.6-2.6) | 13.2 (12.1-14.3) | 13.2 (12.1-14.4) | 4.0 (3.8-4.5) |  | 34.4 (32.9-36.0) | 21.1 (19.8-22.5) | 17.8 (16.6-19.1) | 2.9 (2.4-3.5) | 24.2 (22.8-25.7) | 2.8 (2.3-3.4) | 46.8 (45.2-48.5) | 3.1 (2.6-3.7) |
| SSRI | 23.6 (23.4-23.8) | 20.7 (20.5-20.9) | 0.6 (0.6-0.6) | 1.0 (1.0-1.1) | 6.4 (6.3-6.5) | 10.8 (10.7-11.0) | 5.3 (4.6-5.9) | 0.7 (0.6-0.7) |  | 13.1 (12.9-13.2) | 10.4 (10.3-10.6) | 1.1 (1.1-1.2) | 15.7 (15.5-15.9) | 1.0 (0.9-1.0) | 13.2 (13.1-13.4) | 0.8 (0.7-0.8) |
| SNRI | 25.7 (25.3-26.1) | 23.1 (22.7-23.4) | 0.7 (0.6-0.8) | 1.2 (1.1-1.3) | 7.4 (7.1-7.6) | 11.3 (11.0-11.5) | 6.3 (5.9-7.0) | 1.5 (1.4-1.7) | 49.4 (49.0-49.9) |  | 18.1 (17.8-18.5) | 3.3 (3.2-3.5) | 26.5 (26.1-26.9) | 2.3 (2.1-2.4) | 24.7 (24.3-25.0) | 1.2 (1.1-1.3) |
| TCA | 10.1 (9.8-10.3) | 10.2 (9.9-10.4) | 0.3 (0.2-0.3) | 0.6 (0.6-0.7) | 3.7 (3.6-3.9) | 6.1 (5.9-6.3) | 2.3 (1.9-2.9) | 1.1 (1-1.2) | 32.7 (32.3-33.1) | 15.0 (14.8-15.3) |  | 1.3 (1.2-1.4) | 20.5 (20.2-20.9) | 0.8 (0.7-0.9) | 13.1 (12.8-13.4) | 0.7 (0.6-0.8) |
| Other antidepressants | 21.4 (20.1-22.7) | 22.5 (21.2-23.9) | 1.0 (0.7-1.3) | 1.3 (0.9-1.7) | 10.8 (9.8-11.8) | 12.9 (11.9-14.0) | 6.6 (6.2-7.1) | 2.6 (2.2-3.2) | 51.5 (49.9-53.1) | 40.9 (39.4-42.5) | 19.6 (18.4-20.9) |  | 34.8 (33.3-36.3) | 3.2 (2.7-3.8) | 31.2 (29.7-32.6) | 2.3 (1.9-2.9) |
| Benzodiazepine | 12.2 (12.0-12.5) | 12.0 (11.7-12.2) | 0.6 (0.6-0.7) | 1.5 (1.4-1.6) | 6.8 (6.6-7.0) | 12.1 (11.9-12.4) | 3.3 (2.9-3.7) | 1.3 (1.2-1.4) | 43.3 (42.9-43.6) | 19.3 (19.0-19.6) | 18.0 (17.7-18.3) | 2.1 (2.0-2.2) |  | 1.8 (1.7-1.9) | 19.6 (19.3-19.9) | 1.0 (0.9-1.1) |
| Other anxiolytic | 21.8 (20.3-23.4) | 21.9 (20.4-23.5) | 2.1 (1.6-2.7) | 3.7 (3.0-4.4) | 14.5 (13.2-15.9) | 30.0 (28.2-31.6) | 5.3 (4.7-5.8) | 3.7 (3.0-4.5) | 66.7 (64.9-68.5) | 40.8 (38.9-42.6) | 17.2 (15.8-18.7) | 4.7 (3.9-5.5) | 43.7 (41.8-45.6) |  | 32.3 (30.5-34.1) | 3.6 (2.9-4.3) |
| Z-drug | 19.3 (19.0-19.7) | 18.5 (18.1-18.8) | 0.6(0.5-0.7) | 1.1 (1.0-1.2) | 6.4 (6.2-6.6) | 11.3 (11.0-11.6) | 4.5 (4.0-5.0) | 3.8 (3.6-4.0) | 55.9 (55.4-56.4) | 27.5 (27.1-28.0) | 17.7 (17.3-18.0) | 2.8 (2.7-3.0) | 30 (29.6-30.4) | 2.0 (1.9-2.1) |  | 1.5 (1.4-1.6) |
| Melatonin | 3.9 (3.4-4.5) | 4.3 (3.8-4.8) | 0.2 (0.1-0.3) | 0.4 (0.3-0.6) | 1.8 (1.5-2.1) | 2.9 (2.5-3.3) | 0.9 (0.9-1.0) | 1.8 (1.5-2.2) | 22.6 (21.5-23.6) | 9.4 (8.7-10.1) | 6.5 (5.9-7.2) | 1.5 (1.2-1.8) | 10.9 (10.1-11.7) | 1.6 (1.3-1.9) | 10.5 (9.8-11.3) |  |
|  | Depression diagnosis | Depression symptom | Generalised AD | Panic attack/ disorder | Anxiety disorder other or unspecified diagnosis | Anxiety disorder other or unspecified symptoms | Mixed Depression Anxiety | Insomnia | SSRI | SNRI | TCA | Other antidepressants | Benzo diazepine | Other anxiolytic | Z drug | Melatonin |

Supplementary Table 7: Depression, anxiety and insomnia: relative frequency of treatments and diagnoses/symptoms, according to age (younger than 50yo), with confidence intervals

| Depression-diagnosis |  | 25.7 (25.3-26.2) | 0.6 (0.5-0.7) | 0.8 (0.7-0.8) | 5.6 (5.4 -5.8) | 8.0 (7.7-8.3) | 8.5 (8.1-8.9) | 1.5 (1.4-1.6) | 64.7 (64.3-65.2) | 24.0 (23.6-24.4) | 15.1 (14.8-15.4) | 1.9 (1.8-2.1) | 16.6 (16.3-17.0) | 0.8 (0.7-0.9) | 15.7 (15.4-16.1) | 0.7 (0.6-0.8) |
| --- | --- | --- | --- | --- | --- | --- | --- | --- | --- | --- | --- | --- | --- | --- | --- | --- |
| Depression-symptom | 25.8 (25.3-26.2) |  | 0.4 (0.3-0.5) | 0.7 (0.6-0.8) | 5.3 (5.1-5.5) | 9.1 (8.9-9.4) | 3.9 (3.5-4.6) | 1.9 (1.8-2.1) | 56.4 (55.9-56.8) | 22.1 (21.7-22.5) | 16.5 (16.2-16.9) | 2.0 (1.8-2.1) | 17.9 (17.5-18.2) | 0.8 (0.7-0.8) | 15.4 (15.1-15.8) | 0.8 (0.7-0.9) |
| Generalised AD | 23.4 (21.0-26.1) | 15.1 (13.1-17.4) |  | 3.6 (2.6-4.9) | 18.8 (16.6-21.3) | 22.4 (19.9-24.9) | 7.3 (6.8-7.6) | 0.7 (0.3-1.4) | 51.7 (48.7-54.7) | 27.1 (24.5-29.8) | 16.5 (14.3-18.8) | 2.2 (1.4-3.2) | 31.9 (29.1-34.7) | 2.9 (2.0-4.1) | 17.8 (15.6-20.2) | 1.3 (0.7-2.1) |
| Panic attack/disorder | 16.3 (14.7-18.0) | 14.9 (13.4-16.5) | 2.0 (1.4-2.7) |  | 16.3 (14.7-18.0) | 23.0 (21.2-24.9) | 5.4 (5.0-5.9) | 1.9 (1.4-2.7) | 45.4 (43.2-47.6) | 19.6 (17.9-21.5) | 17.6 (15.9-19.3) | 1.5 (1.0-2.1) | 37.9 (35.8-40.1) | 2.1 (1.5-2.8) | 14.8 (13.3-16.4) | 0.6 (0.3-1.0) |
| Anxiety Disorder other or unspecified-diagnosis | 16.6 (16.0-17.2) | 15.7 (15.1-16.3) | 1.4 (1.2-1.6) | 2.2 (2-2.5) |  | 16.6 (16-17.2) | 6.9 (6.3-7.4) | 3.3 (3.0-3.6) | 44.5 (43.6-45.3) | 19.2 (18.6-19.9) | 15.8 (15.2-16.4) | 2.3 (2.1-2.6) | 30.1 (29.4-30.9) | 1.8 (1.6-2.0) | 15.5 (14.9-16.1) | 0.8 (0.7-1.0) |
| Anxiety Disorder other or unspecified-symptom | 13.1 (12.7-13.5) | 15.0 (14.6-15.4) | 0.9 (0.8-1.1) | 1.8 (1.6-1.9) | 9.2 (8.9-9.6) |  | 3.7 (3.3-4.3) | 1.9 (1.7-2.1) | 43.0 (42.4-43.6) | 17.0 (16.6-17.5) | 15.7 (15.2-16.1) | 1.9 (1.8-2.1) | 30.6 (30.1-31.2) | 1.7 (1.6-1.9) | 16.1 (15.6-16.5) | 0.8 (0.7-1.0) |
| Mixed Depression-Anxiety | 49.4 (48.9-49.8) | 22.9 (22.3-23.1) | 1.1 (1.0-1.3) | 2.2 (2.0-2.4) | 13.6 (13.0-14.0) | 13.2 (12.8-13.8) |  | 5.9 (5.4-6.4) | 61.2 (60.7-61.5) | 25.4 (24.9-26.0) | 15.6 (15.2-16.2) | 2.6 (2.0-3.2) | 20.4 (20.0-20.9) | 0.9 (0.8-1.1) | 16.3 (15.8-16.8) | 0.5 (0.4-0.7) |
| Insomnia | 11.5 (10.7-12.4) | 15.0 (14.1-16.0) | 0.1 (0.1-0.3) | 0.7 (0.5-1.0) | 8.5 (7.8-9.3) | 8.9 (8.2-9.7) | 2.1 (1.9-2.4) |  | 25.8 (24.7-27) | 18.5 (17.5-19.6) | 22.9 (21.8-24.0) | 2.4 (2.0-2.8) | 27.0 (25.8-28.1) | 1.5 (1.2-1.8) | 51.4 (50.1-52.8) | 6.3 (5.6-6.9) |
| SSRI | 17.3 (17.1-17.5) | 15.0 (14.9-15.2) | 0.4 (0.3-0.4) | 0.6 (0.5-0.6) | 4.0 (3.9-4.1) | 7.0 (6.9-7.1) | 2.8 (2.1-3.5) | 0.9 (0.8-0.9) |  | 12.6 (12.4-12.8) | 16.3 (16.1-16.5) | 1.4 (1.4-1.5) | 20.1 (19.9-20.3) | 0.7 (0.7-0.8) | 15.1 (14.9-15.2) | 0.7 (0.7-0.8) |
| SNRI | 16.8 (16.5-17.1) | 15.4 (15.2-15.7) | 0.5 (0.4-0.5) | 0.6 (0.6-0.7) | 4.6 (4.4-4.7) | 7.3 (7.1-7.5) | 3.0 (2.8-3.4) | 1.7 (1.6-1.8) | 33.0 (32.6-33.4) |  | 21.7 (21.3-22.0) | 3.0 (2.8-3.1) | 28.8 (28.5-29.2) | 1.3 (1.3-1.4) | 23.3 (23.0-23.6) | 1.6 (1.5-1.7) |
| TCA | 5.4 (5.3-5.5) | 5.9 (5.8-6.0) | 0.2 (0.1-0.2) | 0.3 (0.3-0.3) | 1.9 (1.8-2.0) | 3.4 (3.3-3.5) | 1.0 (0.9-1.2) | 1.1 (1-1.1) | 21.8 (21.6-22.0) | 11.0 (10.9-11.2) |  | 1.0 (0.9-1.0) | 17.9 (17.7-18.1) | 0.4 (0.3-0.4) | 12.4 (12.2-12.6) | 0.7 (0.6-0.7) |
| Other antidepressants | 12.0 (11.2-12.7) | 12.2 (11.4-13.0) | 0.4 (0.2-0.5) | 0.4 (0.3-0.6) | 4.9 (4.4-5.5) | 7.3 (6.7-8.0) | 2.8 (2.4-3.2) | 1.9 (1.6-2.3) | 33.3 (32.2-34.4) | 26.5 (25.5-27.5) | 16.8 (15.9-17.7) |  | 36.5 (35.3-37.6) | 1.2 (1.0-1.5) | 24.7 (23.7-25.8) | 2.5 (2.1-2.9) |
| Benzodiazepine | 7.5 (7.3-7.6) | 8.0 (7.8-8.2) | 0.4 (0.3-0.4) | 0.8 (0.7-0.8) | 4.6 (4.4-4.7) | 8.4 (8.2-8.6) | 1.7 (1.0-2.0) | 1.6 (1.5-1.6) | 33.8 (33.5-34.1) | 18.5 (18.2-18.7) | 22.5 (22.3-22.8) | 2.6 (2.5-2.7) |  | 0.9 (0.9-1.0) | 19.8 (19.5-20.0) | 1.3 (1.2-1.3) |
| Other anxiolytic | 17.5 (15.8-19.2) | 16.3 (14.7-18.0) | 1.6 (1.1-2.2) | 2.1 (1.5-2.8) | 12.9 (11.5-14.5) | 22.5 (20.7-24.4) | 3.4 (3.0-3.8) | 4.1 (3.3-5.1) | 58.1 (55.7-60.2) | 41.3 (39.1-43.5) | 22.7 (20.9-24.6) | 4.2 (3.4-5.2) | 44.8 (42.6-47.0) |  | 31.9 (29.9-34.0) | 3.2 (2.5-4.0) |
| Z-drug | 10.3 (10.0-10.5) | 10.0 (9.8 -10.3) | 0.3 (0.2-0.3) | 0.5 (0.4-0.5) | 3.4 (3.3-3.6) | 6.4 (6.2-6.6) | 1.8 (1.2-2.5) | 4.3 (4.2-4.5) | 36.8 (36.4-37.2) | 21.7 (21.4-22.0) | 22.7 (22.4-23.1) | 2.6 (2.5-2.7) | 28.8 (28.4-29.1) | 1.0 (0.9-1.0) |  | 2.1 (2.0-2.2) |
| Melatonin | 8.6 (7.7-9.6) | 9.8 (8.8-10.9) | 0.4 (0.2-0.7) | 0.4 (0.2-0.6) | 3.5 (2.9-4.2) | 6.5 (5.7-7.4) | 1.1 (0.9-1.3) | 10.3 (9.3-11.4) | 34.5 (32.9-36.1) | 28.8 (27.2-30.3) | 24.7 (23.3-26.2) | 5.1 (4.3-5.9) | 35.9 (34.3-37.6) | 1.9 (1.5-2.4) | 41.2 (39.5-42.9) |  |
|  | Depression diagnosis | Depression symptom | Generalised AD | Panic attack/ disorder | Anxiety disorder other or unspecified diagnosis | Anxiety disorder other or unspecified symptoms | Mixed Depression Anxiety | Insomnia | SSRI | SNRI | TCA | Other antidepressants | Benzo diazepine | Other anxiolytic | Z drug | Melatonin |

Supplementary Table 8: Depression, anxiety and insomnia: relative frequency of treatments and diagnoses/symptoms, according to age (older than 50yo), with confidence intervals
